# No detectable effect of RNA-binding protein Hfq absence in *Staphylococcus aureus*

**DOI:** 10.1186/1471-2180-7-10

**Published:** 2007-02-09

**Authors:** Chantal Bohn, Candice Rigoulay, Philippe Bouloc

**Affiliations:** 1 Signalisation et Réseaux de Régulations Bactériens. Institut de Génétique et Microbiologie, CNRS/UMR 8621, Centre scientifique d'Orsay, Université Paris Sud, bâtiment 400, 91405 Orsay Cedex, France

## Abstract

**Background:**

The RNA-binding protein Hfq is involved in stress and virulence of several pathogens, probably due to its role as mediator in small RNA (sRNA)-mRNA interactions. In this study, we investigate the function of Hfq in the Gram-positive pathogen *Staphylococcus aureus*, by constructing *hfq *null mutant derivatives.

**Results:**

We report that unexpectedly, in *S. aureus*, Hfq does not seem to play a crucial role in stress response, RNAIII or *spa *mRNA quantity and exoprotein expression, as tested in three virulent genetic backgrounds. Moreover, a global analysis of the RN6390 *hfq *mutant, which tests ~ 2000 phenotypes, supports our results concerning the non-implication of Hfq in stress response, and shows that Hfq is also not involved in resistance to several chemical agents and antibiotics and does not seem to be implicated in metabolic pathways.

**Conclusion:**

Our data suggest that although sRNA-mRNA interactions in *S. aureus *are decisive for gene expression regulation, they do not require the RNA-chaperone protein Hfq. These interactions possibly require an RNA-chaperone protein other than Hfq, which remains to be found.

## Background

The small RNA-binding protein Hfq was first identified in *Escherichia coli *as a required factor for replication of phage Qβ RNA [1]. In *E. coli*, Hfq is a global regulator that modulates the stability, translation and polyadenylation of numerous mRNAs (for review, see [2]). It is proposed that Hfq acts as a RNA chaperone protein by mediating interactions between many regulatory small RNAs (sRNAs) and their mRNAs targets (for review, see [2,3]). Inactivation of the *hfq *gene in *E. coli *results in lower growth and sensitivity to various environmental stresses [4,5]. Genome analyses show that Hfq-like proteins are conserved in many (but not all) Gram-negative and -positive bacteria [6]. Hfq is involved in stress response and/or virulence of several Gram-negative pathogens (*Vibrio cholerae*, *Brucella abortus*, *Yersinia enterocolitica*, *Pseudomonas aeruginosa, Shigella flexneri, Salmonella enterica *[7-13] and the Gram-positive pathogen, *Listeria monocytogenes *[14].

*Staphylococcus aureus *is a Gram-positive pathogen responsible for a wide variety of human infections, ranging from superficial skin and wound infections to deep abscesses (endocarditis and meningitis), septicemia, or toxin-associated syndromes (e.g. food poisoning and toxic shock syndrome) [15]. *S. aureus *is also a leading cause of hospital and community-acquired infections whose treatment is becoming increasingly difficult due to the emergence of multiple antibiotic resistance determinants [16]. The type of infection caused by *S. aureus *depends on virulence factors and stress response pathways present in infectious strains, and reflects the coordinated action of numerous regulators (for review, see [17]). The best-characterized regulator of virulence gene expression is the *agr *(accessory gene regulator) quorum sensing system (for review, see [17]), which comprises two divergent transcripts, RNAII and RNAIII. The small RNAIII transcript (514 pb) is a key regulator that modulates production of *S. aureus *extracellular proteins at the transcriptional and post-transcriptional level (for review, see [17]).

*S. aureus *encodes one Hfq-like protein (8.9 kDa), whose structure has been described [18]. Under *in vitro *conditions, it specifically binds RNAIII and *spa *mRNA [19]. More recent studies revealed the expression of other regulatory sRNAs among *S. aureus *infectious strains [20-22], which may be involved in the regulation of virulence. These observations suggest that RNA-RNA interactions are decisive for virulence regulation in *S. aureus*, while the role of Hfq in these interactions remains to be elucidated.

In this study, we constructed *hfq *derivatives in three *S. aureus *backgrounds that differ in their virulence capacities (RN6390, COL and Newman; [23]), and compared them to their respective WT strains, in order to determine the Hfq function(s). In the experimental conditions and the genetic backgrounds tested, Hfq does not display any detectable role in stress tolerance, RNA stability or exoprotein expression. To provide a more complete analysis of the *hfq *associated phenotypes, we also compared the RN6390 WT and *hfq *mutant strains using the Phenotype Microarray (PM) Technology, which tests ~ 2000 phenotypes simultaneously (including utilization of several sources of carbon, nitrogen, phosphate and sulfur, and sensitivity to different stresses and chemical agents). Based on our results, we can postulate that in *S. aureus*, RNA-RNA interactions do probably not require the involvement of RNA chaperone proteins, or that at least a protein other than Hfq, as yet unidentified, is responsible for the modulation of RNA interactions.

## Results and discussion

### Inactivation and expression of *hfq *in *S. aureus*

**Figure 1:**
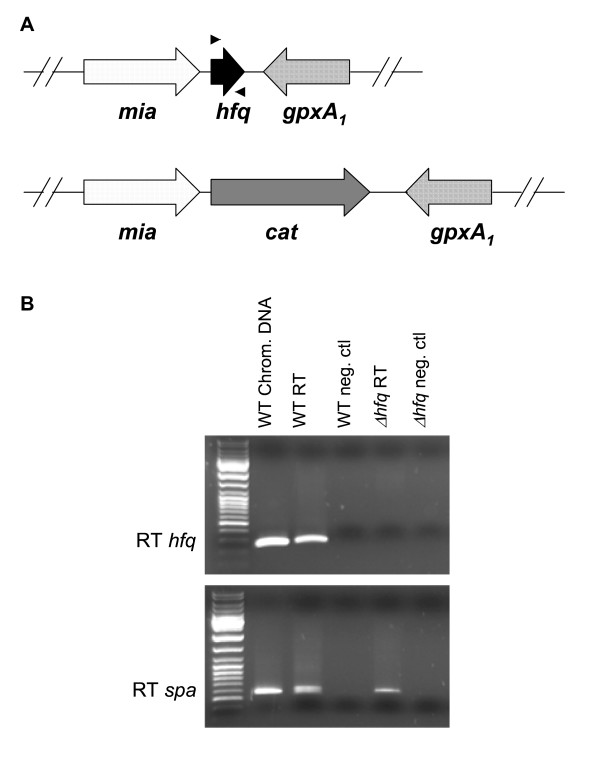
**Inactivation of *hfq *in *S. aureus *(1A) and expression of *hfq *in *S. aureus *RN6390 (1B)**. The complete *hfq *ORF is removed and replaced by the *cat *gene (1A). Genes directly up-(*mia*) and downstream (*gpxA*_1_) of *hfq *are shown, and primers used for RT-PCR experiments are indicated by arrows. Positions of ORFs related to the N315 annotated genome are respectively: *mia *(13022047-1302982); *hfq *(1302997-1303230), *gpxA*_1 _(c1303928-1303452). Total RNAs from post-exponential growth phase cultures of RN6390 WT and *hfq *mutant strains were first retro-transcribed using random hexamer primers. PCR amplification specific to the *hfq *ORF was realized from the cDNAs, obtained from random retro-transcription (RT) (1B). Chromosomal DNA of WT (WT chrom. DNA) was used as positive control of PCR amplification. PCR amplification of the *spa *gene was also used as a positive control of cDNA quality from the RN6390 *hfq *strain. Negative controls of RT-PCR reactions were also shown (neg. ctl).

We first constructed an *hfq *mutant in the *S. aureus *avirulent strain RN4220, in which the region corresponding to the *hfq *ORF was replaced by a cassette conferring Cm resistance (see Methods and Fig. 1A). Gene inactivation was verified by Southern blot (data not shown) and the *hfq *mutation was then transferred by phage transduction into three virulent strains that differ in their virulence regulation pathways, RN6390, Newman and COL. Gene transfer was confirmed by PCR and RT-PCR (Fig. 1B).

In *E. coli*, Hfq is one of the most abundant intracellular proteins, whose level in exponential growth phase is reportedly around 30,000 to 60,000 molecules per cell [24]. Hfq levels further increase during the transition to stationary phase [25]. These data suggest that Hfq likely plays a crucial role in controlling expression of numerous growth-related genes. In view of the described properties of Hfq, we asked whether *hfq *expression levels in *S. aureus *were as high as in *E. coli*. We first followed *hfq *expression during bacterial growth in BHI medium, by northern blot experiments, using an *hfq*-specific fragment as probe. No transcript corresponding to *hfq *mRNA was detected in RN6390, Newman or COL WT backgrounds (from total RNAs extracted from both exponential and stationary phase cultures; data not shown). In these experiments, an *E. coli hfq *strain containing a plasmid carrying the *S. aureus hfq *gene under control of a tetracycline inducible promoter system was used as positive control of *hfq *mRNA expression. We then compared *hfq *expression using RT-PCR, which is a more sensitive detection method than northern blotting. Steady state levels obtained from RN6390 WT and *hfq *post-exponential RNA extracts are represented on Figure 1B. A band corresponding in size to the *hfq *amplicon (which is comparable in size to the fragment amplified from chromosomal DNA) was detected in the WT profile, suggesting that *hfq *is expressed in *S. aureus*. As expected, no amplification from both *hfq *mutant strain chromosomal DNA (not shown) and cDNAs (Fig. 1B) was observed, which confirms the *hfq *ORF deletion. Positive amplification from cDNAs of the RN6390 *hfq *strain was visualized with PCR using *spa *specific primers, confirming the quality of these extracts (Fig. 1B). Similar results were obtained with RT-PCR experiments realized with Newman strain.

These results indicate that *hfq *gene is expressed in *S. aureus *in the experimental conditions tested but at a very low level, as no transcript could be visualized by northern blot experiments. In view of these data, we speculate that Hfq protein is probably less abundant in *S. aureus *than in *E. coli*.

### *hfq *inactivation has no effects on growth and stress resistance

We first compared growth of *hfq *mutants of RN6390, COL and Newman, with their respective WT strains in aerated BHI rich medium or in aerated RPMI synthetic medium at 37°C. No growth differences were noticed in any of the genetic backgrounds tested (data not shown) in either media. As Hfq is reportedly involved in stress tolerance in many pathogens [8,10,14], we also tested the ability of staphylococcal *hfq *mutants to resist to several environmental stresses: Under osmotic (NaCl), oxidative stress (H_2_O_2_) or heat-shock (44°C) conditions, the *hfq *mutant grew as well as the WT strain in all genetic backgrounds (data not shown). In *L. monocytogenes*, the contribution of Hfq in stress response depends on the alternative sigma factor σ^B ^pathway, as σ^B ^controls *hfq *stress-inducible transcription [14]. In *S. aureus*, Hfq does not seem to be implicated in stress resistance, regardless of the presence or absence of the σ^B ^pathway, as the same results were obtained in both σ^B ^pathway functional (COL, Newman) and defective (RN6390) strains.

### Hfq does not affect RNAIII or *spa* mRNA quantity *in vivo*

**Figure 2:**
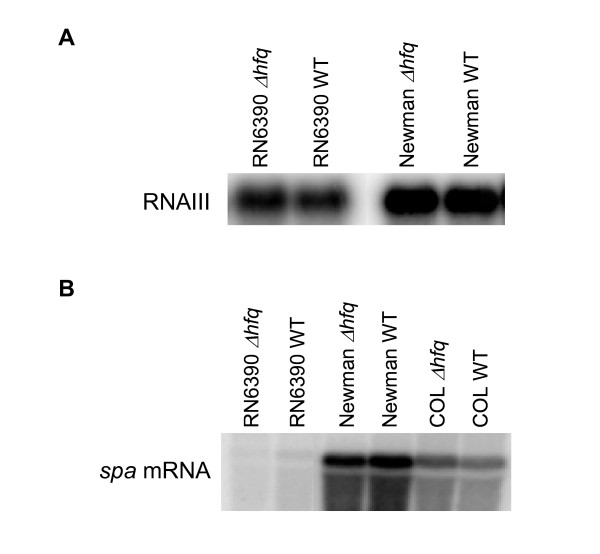
**Northern blot analysis of RNAIII (2A) or *spa *mRNA (2B) expression in *S. aureus *WT and *Δhfq *strains**. Total RNA was extracted from post-exponential phase cultures of RN6390, Newman and COL, WT and *Δhfq *strains. 15 μg of total RNA of RN6390 and Newman strains were analysed by northern blot, using an RNAIII-specific probe (2A). Total RNA from RN6390, Newman and COL strains was also compared for *spa *mRNA expression, using a *spa*-specific probe (2B). Results from three independent experiments were identical.

*In vitro *studies showed that Hfq specifically binds the *spa *mRNA regulatory region, and more tightly, RNAIII [19]. *spa *encodes the Staphylococcal protein A (Spa), a major surface protein. Its expression is known to be repressed by RNAIII at the transcriptional level by an undetermined mechanism, but additional studies have shown that RNAIII also inhibits *spa *expression by affecting its translation and stability [19]. To elucidate the involvement of Hfq in the stability of RNAIII and *spa *mRNA *in vivo*, we compared their steady state levels in WT and *hfq *mutant strains. No significant differences were observed in RNAIII expression in the *hfq *mutant, compared to the WT strain, whether in the RN6390 or Newman context (Fig. 2A). When we compared *spa *RNA transcript profiles in RN6390, Newman and COL backgrounds, no differences between *Δhfq *and WT were detected (Fig. 2B). Variation of *spa *mRNA expression visualized between strain backgrounds reflects differences in virulence regulatory pathways, as previously described [23]. Amounts of protein A were also analysed in staphylococcal strains by western blot experiments [26] using a monoclonal anti-protein A antibody (Sigma, Saint Louis, MO, USA); again no differences were observed (data not shown). The results described above show that Hfq does not affect the quantities of RNAIII or of *spa *mRNA *in vivo*. In view of these observations, we speculate that in the natural context of the bacterium, Hfq does not contribute to the quantities of RNAIII and *spa *mRNA and likely has no effect on the formation of the RNAIII-*spa *mRNA complex. This is relevant to previous *in vitro *results showing that increasing concentrations of Hfq have no effect on the formation of RNAIII-*spa *mRNA complex [19].

### Hfq does not affect exoprotein expression profiles

*S. aureus *secretes numerous virulence factors, which are mainly produced during the post-exponential phase, and whose expression involves the coordination of many regulators, including *agr *RNAIII (for review, see [17]). To test the possible role of Hfq in the production of secreted virulence factors, we compared exoprotein profiles in RN6390, Newman, and COL WT strains and their *Δhfq *derivatives. No differences were observed in the various genetic backgrounds tested (data not shown). Hemolysins and proteases are major secreted virulence factors in some *S. aureus *virulent strains, such as RN6390. To test the effect of Hfq on production of those secreted enzymes, we compared hemolytic and proteolytic activities in RN6390 and Newman WT and *hfq *mutant strains. Stationary cultures were streaked onto BHI medium containing rabbit blood (which reveals α-hemolytic activity), sheep blood (corresponding to β-hemolytic activity) and horse blood (δ-hemolytic activity), or onto NB medium containing skimmed milk (to reveal proteolytic activities). No differences were observed in halos corresponding to the different enzymatic activities in the two genetic backgrounds tested (data not shown), which suggests that the lack of Hfq does not affect production of either hemolysins or secreted proteases.

Altogether, these results suggest that deletion of *hfq *does not affect exoprotein expression profiles. It is thus likely that Hfq is not decisive for the production of secreted virulence factors.

### PM analysis of RN6390 *hfq *mutant

**Figure 3:**
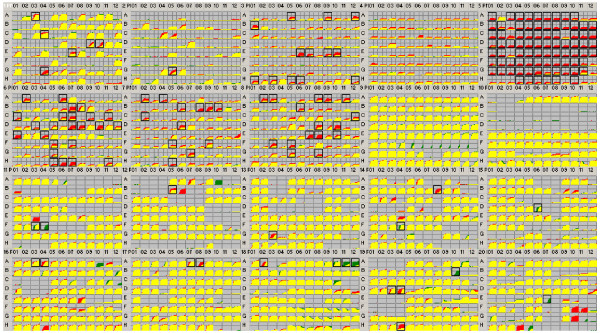
**PM comparative analysis of RN6390 WT and *hfq *strains**. The WT and the *hfq *mutant were subject to PM analysis of ~ 2000 phenotypes. Incubation and time course curves for respiration (tetrazolium color formation) in specific conditions were generated with Omnilog-PM software (see for more details [42]). The PM kinetics shows consensus data comparing the RN6390 *hfq *mutant (green) to its respective WT strain (red). Red indicates a stronger response by the parental strain and green indicates a stronger response by the *hfq *mutant. When the two strains have equivalent metabolism or sensitivity to a stress or chemical agent, the red and green kinetic graphs overlap and are yellow. Boxes surrounding a specific condition indicate a significant difference in response. Values of gain or loss of phenotypes are in Table 1 of supplementary data.

PM assay is a relatively new technology in which ~ 2000 phenotypes are tested simultaneously, and has already been used for comparative analysis between strains in several bacterial species [27-29]. Strains are grown in microtiter plate, each well containing different media. This technology may help to determine gene functions. Respiration is used in PM as an indication of bacterial growth, and was compared between strains in media containing different sources of carbon (PM1 to PM2), nitrogen (PM3 and PM6 to PM8), phosphorus and sulfur (PM4), nutrients or cofactors (PM5), or in the presence of different stresses or chemical agents (PM9 to PM20). Consensus PM results of comparative analysis of RN6390 WT and *hfq *mutant strains are shown on Figure 3. Additional file 1 indicates values of phenotypes gained or lost. Briefly, slight or no differences between strains were observed under any metabolic conditions, and differences in sensitivity to chemical agents observed could not be reproduced by independent experimentation.

Metabolism tests (PM1 toPM8), performed in a synthetic medium, showed negative differences for utilization of several amino acids or dipeptides as nitrogen source (PM3 and PM6 to PM8); although the signal decreases were very weak. These data seemed to suggest that the *hfq *mutant strain could be defective for nitrogen metabolism. To test this hypothesis, we compared growth of RN6390 WT and *hfq *mutant strains in a synthetic complete medium or in a synthetic minimal medium (containing the essential amino acids for auxotrophy, glutamate and leucine; Elise Durant, INRA, personal communication) with or without several nitrogen sources, including different combinations of amino acids (including glutamate and/or aspartate) as used in the PM assay. No differences in growth were observed between the two strains (data not shown), suggesting that the *hfq *mutant uses different nitrogen sources as well as the WT strain. The weak differences detected by PM analysis (PM3, PM6 to PM8) may reflect the use in PM of a standard chemically minimal medium that lacks the essential amino acids, where only residual growth of both strains occurred. In this case, it is likely that phenotypes observed result from residual respiration of surviving bacteria in the medium and not from real bacterial growth.

Stress or chemical agent sensitivity PM tests (PM9 to PM20) were performed in rich medium, similar to LB. Essentially no differences were observed in osmotic (PM9) and pH (PM10) stress panels between the RN6390 WT and *hfq *mutant strains (Fig. 3), which confirmed some results obtained by conventional stress tests (see above). Positive or negative differences were observed for some chemical agents or antibiotics (PM11 to PM20; see Fig. 3, and Additional file 1) but signals were weak (see Additional file 1). The only real signal concerned Cm resistance of the *hfq *mutant, a consequence of the strain construction (PM11, F3-4 and PM 14, F4, and derivative thiamphenicol, PM18, A10-12). We compared sensitivity of some chemical agents or antibiotics that gave negative responses (cobalt chloride, PM13, G3; cefotaxime, PM16, A3-4), or positive signals (domiphen bromide, PM15, D6; dodine, PM20, E7) on MH broth by measuring the MIC or on agar plates using disk-specific antibiotics. Again, no significant differences were observed between WT and *hfq *mutant strains (data not shown).

This comparative analysis showed that among ~ 2000 phenotypes, essentially no reproducible phenotypic differences between *S. aureus *RN6390 WT and its *hfq *derivative mutant were detected. For metabolic tests, the few phenotypes that were noted likely have no physiological significance, and may result from the poor growth of both strains. We thus conclude that Hfq has no role on stress response and on chemical agent sensitivity and probably no impact on metabolic pathways in *S. aureus*.

## Conclusion

In this study, we tried to elucidate the roles of the RNA-binding protein Hfq in *S. aureus *cell physiology and regulation. Our results indicate that Hfq does not seem to contribute to the adaptation to stress responses, RNAIII or *spa *mRNA levels, or to general exoprotein production in *S. aureus*. Some of these data have been recently suggested [30]. A more general approach, which tests ~ 2000 phenotypes simultaneously confirmed our observations (e.g., no implication of Hfq in stress sensitivity) and also showed that Hfq has likely no impact on metabolic pathways or on resistance to different chemical agents or antibiotics. Nevertheless, it remains possible that Hfq has a role in environmental conditions that differ from those tested; for example, those encountered to proliferate inside the host during the infection process. We compared growth in rabbit serum between a wild type and its *hfq *derivative mutant, in the three different genetic contexts; no differences were observed (data not shown). This result suggests that Hfq may not be crucial for survival inside the host.

In *E. coli*, Hfq contributes to interactions between regulatory sRNAs and their mRNA targets (for review, see [2]). It also forms a ribonucleoprotein complex with RNase E that affects mRNAs turnover [31]. Staphylococcal genome analyses indicate the lack of an RNase E-like protein. In *Bacillus subtilis*, RNases J1 and J2 have functional homologies with RNase E but no sequence similarity [32,33]; homolog of these RNases are present in *S. aureus*. However, it has never been demonstrated that RNases J1 and J2 functions are dependant of Hfq. Although RNA-RNA interactions appear to be decisive for regulatory pathways in *S. aureus*, regulation mechanisms are likely to occur independently of Hfq and RNase E.

Searches for Hfq conservation through bacterial species have shown that Hfq homologs are absent in several low GC Gram-positive bacteria, such as *Lactococcus lactis*, *Streptococcus pneumoniae*, and *Streptococcus pyogenes *[6]. In *Borrelia burgdoferi*, genome analyses reveal the lack of both Hfq and RNase E proteins [34]. We propose that in *S. aureus*, under conditions known to involve Hfq in other bacteria, RNA-RNA interactions do not require Hfq. An as yet uncharacterized RNA-chaperone protein might be responsible for the modulation of RNA interactions in this bacterium. Database searches in *S. aureus *genomes for Sm-like domains, which are characteristic of RNA-binding protein like Hfq, reveal no other candidates than Hfq. If *S. aureus *sRNAs are important for mRNA turnover, we suggest the existence of alternative mechanism that substitute for Hfq and RNase E that remains to be discovered.

## Methods

### Bacterial strains, plasmids and growth conditions

Bacterial strains and plasmids used in this study are listed in Table 1. *S. aureus *strains were routinely grown in BHI medium with aeration at 37°C, or at 30°C for the RN4220 strain harboring the thermosensitive plasmid. Defined synthetic medium used for staphylococcal growth was either RPMI medium 1640 (Gibco-Invitrogen, Eugene, USA) or as published [35]. *E. coli *DH5αZ1 [36], which was used for cloning experiments, was grown in LB broth at 37°C. Antibiotics were added to media as needed at the following concentrations: Cm, 5 μg/ml for *S. aureus *and 10 μg/ml for *E. coli*; Ery, 2 μg/ml for *S. aureus *and Amp, 100 μg/ml for *E. coli*. X-Gal was added as needed to BHI plates at a final concentration of 150 μg/ml.

For stress studies, staphylococcal cultures were prepared as follows: serial dilutions of exponential cultures were spotted on BHI plates containing or not an osmotic stress agent (NaCl, 0.5 to 2 M) or an oxidative stress agent (H_2_O_2_, 1 to 2 mM), and incubated at 37°C for 24 h. Growth at high temperature was examined by plating cells on BHI medium and incubating at 44°C for 24 h.

### DNA manipulations

Plasmid and chromosomal DNA preparations, PCR amplifications, and DNA modifications were performed according to commonly used techniques or suppliers' instructions. Lysis of *S. aureus *cell suspensions was achieved by treatment with 100 μg/ml of lysostaphin (Sigma-Aldrich, St. Louis, MO, USA) for 1 hour at 37°C, followed by standard methods for DNA preparation. DNA transformation for *E. coli *[37] and for *S. aureus *[23] was performed as described.

### Chromosomal deletion of *hfq*

*hfq *was inactivated using the pMAD plasmid, which is thermosensitive for replication [38], as described [23]. Briefly, regions up- and downstream of *hfq *were amplified, fused to flank the pC194 *cat *amplified gene [39] (see primer list in Table 2) and cloned into the pGEM-T-Easy vector (Promega, Madison, WI, USA). The segment of interest was then subject to appropriate digestions and cloned into pMAD. The resulting plasmid, pMAD *Δhfq*::*cat *was introduced into the RN4220 strain and *hfq *mutant derivatives were selected as described [23]. Gene inactivation was confirmed by Southern blotting [26], after HaeIII digestion of RN4220 WT and *hfq *chromosomal DNA, using a PCR product corresponding to the Hfq ORF region as a probe (see Table 2 for primers used). The *hfq *mutation was then transferred into RN6390, Newman and COL *S. aureus *strains by ϕ11 phage-mediated transduction [23]. Mutation transfer was confirmed by PCR and RT-PCR (see primers list in Table 2).

### RNA manipulations

Overnight cultures of staphylococcal strains in BHI medium were diluted 1000-fold in the same medium and grown at 37°C. Total RNAs of mid-or post- exponential or stationary phase cultures were extracted as previously described [40] or using the RNA spin mini Kit (GE Healthcare, Buckinghamshire, UK). Northern blot experiments were performed using *hfq*, RNAIII- and *spa *specific fragments as probes (see Table 2 for primers list), which were α^32^-P labelled, as described [26]. RNA transcripts were quantified, using *hu *mRNA as positive control of constitutive expression. For *hfq *expression studies, random RT-PCR experiments were performed using the Thermoscript RT-PCR System (Invitrogen, Eugene, USA); primers used for successive PCR are in Table 2.

### Protein extract preparation, protein profile analysis and determinations of extra-cellular enzymatic activities

Overnight cultures of staphylococcal strains in BHI medium were diluted 100-fold in the same medium and grown at 37°C. Supernatant protein extracts from post-exponential or stationary phase cultures were prepared as described [23] and protein profiles were compared by PAGE after staining with Coomassie blue reagent [26]. For hemolytic or proteolytic activities, staphylococcal stationary phase cultures were streaked on BHI medium containing 5% rabbit, sheep, or horse blood, or on NB medium containing 10% skimmed milk. Enzymatic halos were compared after overnight growth at 37°C.

### PM analysis

*S. aureus *RN6390 WT and *hfq *mutant strains were subject to full profile (20 panels) PM analysis, as described elsewhere [28,29,41]. This extensive analysis contains ~ 2000 tests which include utilization of different sources of carbon (PM1 and PM2; ~ 200 tests), nitrogen (PM3 and PM6 to PM8; ~ 400 tests) phosphorus and sulfur (PM4; ~ 100 tests), nutrient stimulation (PM5; ~ 100 tests) as well as sensitivity to several stresses (PM9 and PM10; ~ 200 tests) and to chemical agents that affect various biological pathways (PM11 to PM20; ~ 1000 tests). Detailed information of PM experimental conditions is available [42]. Briefly, respiration during bacterial growth under different conditions is monitored by NADH production, measured with a tetrazolium dye and evaluated for intensity of color formation. Phenotypic data were recorded over a 24-hour period. Differences in measurements of WT and *hfq *mutant strains are calculated. The WT strain is recorded as a red tracing, and the *hfq *mutant as a green tracing. Areas of overlap (no changes) between the two measurements are colored in yellow, whereas differences are colored in red or green for WT or *hfq *mutant respectively. The consensus profile presented is the result of two independent comparative analyses.

To confirm some of the observations with the nitrogen metabolic arrays (PM3 and PM6 to PM8), growth of RN6390 WT and *hfq *mutant strains was compared in a chemically defined medium [35] containing or not different sources of nitrogen. Sensitivity to selected chemical agents (cobalt chloride, dodine, domiphen bromide) or antibiotics (cefotaxime) was tested by determining the Minimum Inhibitory Concentration, (MIC) or by using the agar disk diffusion method (Cefotaxime, Fluka). Both of these experiments were performed in Mueller Hinton broth.

## Abbreviations

WT: Wild Type; BHI: Brain Heart Infusion; RPMI: Roswell Park Memorial Institute; LB: Luria-Bertani; NB: Nutrient Broth; MH: Mueller Hinton; Ery: Erythromycin; Cm: Chloramphenicol; Amp: Ampicillin; X-Gal: 5 bromo-4-chloro-3-indolyl-β-D-galactopyranoside; *spa*: Staphylococcal protein A gene; ORF: Open Reading Frame.

## Authors' contributions

CB carried out the genetic and RNA experiments. CR carried out the physiological and protein experiments. All the authors contributed to analysis and interpretation of data. CR and PB drafted the manuscript. All authors read and approved the final manuscript.

## Supplementary Material

Additional file 1 PM Analysis Results. This table indicates values of phenotypes gained or lost. Click here for file

## Figures and Tables

**Table 1:** Strains and plasmids used in this study.

	**Key features**	**Reference**
**Strains**		

*E. coli *DH5αZ1	Δ(*argF-lac*)169 ϕ80*Δlac*Z58(M15) *gln*V44 (AS) *rfb*D1 *gyr*A96 (Nal^R^) *rec*A1 *end*A1 *spo*T1 *thi*-1 *hsdR1*7 Z1(*lacR tetR *Sp^R^)	[36]
*S. aureus *RN4220	Avirulent mutant of NCTC 8325-4 that accepts foreign DNA	[43]
S. aureus RN6390	Laboratory virulent strain derived from NCTC 8325	[43]
*S. aureus *COL	Highly methicillin resistant clinical isolate	[44]
*S. aureus *Newman	Virulent strain containing a high level of Clumping Factor	[45]
*S. aureus *RN4220 *hfq*	RN4220, *Δhfq*::*cat*, Cm^R^	This work
*S. aureus *RN6390 *hfq*	RN6390, *Δhfq*::*cat*, Cm^R^	This work
*S. aureus *COL *hfq*	COL, *Δhfq*::*cat*, Cm^R^	This work
*S. aureus *Newman *hfq*	Newman, *Δhfq*::*cat*, Cm^R^	This work

**Plasmids**		

PMAD	Thermosensitive plasmid in *S. aureus *derived from pRN5101 and containing the *bgaB *gene, Ery^R^, Amp^R^	[38]
pMAD*Δhfq*::*cat*	pMAD containing up- and downstream regions from *hfq *gene cloned on either side of the Cm cassette (*cat*); used for construction of the *S. aureus *RN4220 *hfq *mutant, Ery^R^, Amp^R^, Cm^R^, *bgaB*	This work

**Table 2:** Primers used in this study*.

**Name**	**Position**	**Sequence**	**Use**
SA-*hfq *5up (BamH1)	F	5'CGGGATCCATGACGATTCTTTAGGTGA3'	Used for cloning upstream region of *hfq*
SA-*hfq *5down (KpnI)	R	5'GGGGTACCTGTCGGACTCCTTTTACT3'	
SA-*hfq *3up (SmaI)	F	5'TCCCCCGGGATGGGCACGATTTAATGAC3'	Used for cloning downstream region of *hfq*
SA-*hfq *3down (EcoRI)	R	5'CGGAATTCTACTTGAAACACCTGATT	
SA-*cat *5 (KpnI)	F	5'GGGGTACCGCACAGACAGGACAAAATCG	Used for *cat *amplification
SA-*cat *3 (SmaI)	R	5'TCCCCCGGGTTCCGAGGCTCAACGTC3'	
SA-*hfq *verif up	F	5'ATCATAGCAGGTGGAACAG3'	Used for verification of *hfq *deletion
SA-*hfq *verif down	R	5'CGAAAGAGAAATAGAAAAAT3'	
SA-RNAIII 5	F	5'CAGAGATGTGATGGAAAATAGTTG3'	Used for RNA III probe amplification
SA-RNAIII 3	R	5'ATTAAGGGAATGTTTTACAGTTATT3'	
SA-*spa *5	F	5'TTAGCATCTGCATGGTTTGC3'	Used for *spa *probe amplification
SA-*spa *3	R	5'ACAACGTAACGGCTTCATCC3'	
SA-*hu *5	F	5'AGATTTAATCAATGCAGTTGCAGA3'	Used for *hu *probe amplification
SA-*hu *3	R	5'AATGCTTTACCAGCTTTG AATGCT3'	
SA-*hfq *5 (NdeI)	F	5'GGAATTCCATATGATTGCAAACGAAAAC3'	Used for *hfq *probe amplification, RT-PCR
SA-*hfq *3 (HindIII)	R	5'CCCAAGCTTATTCTTCACTTTCAGTAG3'	

*F: forward; R: reverse. Restriction enzyme sites are underlined.
