# Supplementary material for: No detectable effect of RNA-binding protein Hfq absence in Staphylococcus aureus
Source: BMC Microbiol. 2007 Feb 9;7:10. doi: 10.1186/1471-2180-7-10 (PMC1800855; doi:10.1186/1471-2180-7-10)
Supplement: Additional file 1 — PM Analysis Results. This table indicates values of phenotypes gained or lost. [file 1471-2180-7-10-S1.pdf]

PHENOTYPE MICROARRAY (PM)- NO	WELL	VALUE	SUBSTRATE	MODE OF ACTION
**Phenotypes Gained:**				
PM20B	E07	95	Dodine	fungicide, guanidine, membrane permeability
PM15B	D06	74	Domiphen bromide	membrane, detergent, cationic, fungiside
PM18C	A10,A11,A12	434	Thiamphenicol	protein synthesis
PM11C	F03,F04	212	Chloramphenicol	protein synthesis
PM14A	F04	61	Chloramphenicol	protein synthesis
PM19	B10	130	2,4-Dintrophenol	respiration, ionophore, H+
**Phenotypes Lost:**				
PM17A	A07,A08	-150	b-Chloro-L-Alanine	aa analog, alanine, aminotransferase inhibitor
PM18C	A02	-87	Ketoprofen	anti-capsule
PM01	G04	-142	L-Threonine	C-source
PM02A	G05	-105	Glycine	C-source
PM01	D10	-78	Lactulose	C-source
PM01	A03	-75	N-Acetyl-D-Glucosamine	C-source
PM01	D09	-70	a-D-Lactose	C-source
PM01	E07	-66	a-Hydroxy-Butyric Acid	C-source
PM01	C04	-54	D-Ribose	C-source
PM14A	B08	-126	Fusaric Acid	chelator, lipophilic
PM19	H04	-172	Hexaminecobalt (III) Chloride	DNA synthesis
PM19	D03,D04	-184	Disulphiram	fungicide
PM06	B07	-175	Arg-Asp	N-source
PM08	E08	-161	Val-Glu	N-source
PM06	D07	-155	Glu-Asp	N-source
PM08	D10	-154	Ser-Glu	N-source
PM06	D10	-146	Glu-Ser	N-source
PM07	B09	-145	Met-Gln	N-source
PM03B	E06	-143	Glucuronamide	N-source
PM06	H07	-141	Leu-Glu	N-source
PM03B	E10	-138	D-Mannosamine	N-source
PM08	E07	-136	Val-Gln	N-source
PM06	D08	-134	Glu-Glu	N-source
PM06	E01	-132	Glu-Val	N-source
PM06	H06	-125	Leu-Asp	N-source
PM06	A06	-123	Ala-Glu	N-source
PM08	D08	-122	Ser-Asp	N-source
PM06	C07	-118	Asn-Glu	N-source
PM08	A09	-117	Asp-Gln	N-source
PM08	F03	-113	b-Ala-Gly	N-source
PM07	B08	-105	Met-Asp	N-source
PM07	D06	-104	Pro-Gln	N-source
PM03B	H01	-103	Ala-Asp	N-source
PM06	A02	-103	L-Glutamine	N-source
PM08	A02	-101	L-Glutamine	N-source
PM03B	B01	-99	L-Glutamine	N-source
PM07	G07	-99	Tyr-Glu	N-source
PM07	A02	-99	L-Glutamine	N-source
PM07	B10	-96	Met-Glu	N-source
PM03B	A12	-96	L-Glutamic Acid	N-source
PM03B	E07	-94	D,L-Lactamide	N-source
PM06	E04	-89	Gly-Cys	N-source
PM03B	A05	-89	Urea	N-source
PM08	A11	-89	Glu-Ala	N-source
PM08	A08	-88	Asp-Ala	N-source
PM08	F05	-87	Met-b-Ala	N-source
PM06	F05	-82	His-Asp	N-source
PM03B	A09	-82	L-Asparagine	N-source
PM06	C09	-78	Asp-Asp	N-source
PM08	A03	-77	Ala-Asp	N-source
PM03B	H03	-77	Ala-Glu	N-source
PM03B	E09	-75	D-Galactosamine	N-source
PM06	H11	-75	Leu-Met	N-source
PM08	B06	-75	Ile-Asn	N-source
PM06	F07	-72	His-Leu	N-source
PM06	H05	-72	Leu-Arg	N-source

PHENOTYPE MICROARRAY (PM)- NO	WELL	VALUE	SUBSTRATE	MODE OF ACTION
**Phenotypes Lost(cont'd):**				
PM07	B06	-70	Lys-Val	N-source
PM08	G08	-69	Leu-b-Ala	N-source
PM06	B08	-66	Arg-Gln	N-source
PM07	C02	-65	Met-Leu	N-source
PM07	A04	-65	Leu-Trp	N-source
PM07	F09	-62	Trp-Glu	N-source
PM06	G05	-61	Ile-Gln	N-source
PM06	D03	-60	Asp-Val	N-source
PM07	H05	-60	Val-Asp	N-source
PM08	G05	-59	Gly-D-Ser	N-source
PM06	D12	-59	Glu-Tyr	N-source
PM06	C06	-57	Arg-Val	N-source
PM08	E10	-57	Val-Met	N-source
PM03B	H12	-56	Met-Ala	N-source
PM08	C12	-54	Pro-Asn	N-source
PM06	C01	-53	Arg-Met	N-source
PM03B	H06	-52	Ala-Leu	N-source
PM06	D05	-51	Gln-Gln	N-source
PM07	F05	-51	Thr-Pro	N-source
PM03B	H05	-51	Ala-His	N-source
PM05	B10	-125	Guanine	nutrient stimulation
PM05	G06	-118	Inosine + Thiamine	nutrient stimulation
PM05	C10	-116	Hypoxanthine	nutrient stimulation
PM05	C12	-115	2`-Deoxy-Inosine	nutrient stimulation
PM05	B12	-110	2`-Deoxy-Guanosine	nutrient stimulation
PM05	C11	-104	Inosine	nutrient stimulation
PM05	G11	-101	Menadione	nutrient stimulation
PM05	D05	-100	L-Homoserine Lactone	nutrient stimulation
PM05	D03	-93	Chorismic Acid	nutrient stimulation
PM05	A06	-91	L-Aspartic Acid	nutrient stimulation
PM05	B11	-91	Guanosine	nutrient stimulation
PM05	A08	-90	L-Glutamic Acid	nutrient stimulation
PM05	A04	-88	L-Arginine	nutrient stimulation
PM05	G10	-88	Pyrrolo-Quinoline Quinone	nutrient stimulation
PM05	E10	-88	Uracil	nutrient stimulation
PM05	B09	-85	Guanosine-3',5'-Cyclic Monophosphate	nutrient stimulation
PM05	C06	-85	L-Valine	nutrient stimulation
PM05	A07	-83	L-Cysteine	nutrient stimulation
PM05	A05	-83	L-Asparagine	nutrient stimulation
PM05	D06	-83	D-Alanine	nutrient stimulation
PM05	C05	-82	L-Tyrosine	nutrient stimulation
PM05	D04	-81	Shikimic Acid (-)	nutrient stimulation
PM05	E11	-80	Uridine	nutrient stimulation
PM05	C07	-80	L-Isoleucine + L-Valine	nutrient stimulation
PM05	E06	-80	Pyridoxamine	nutrient stimulation
PM05	D08	-79	D-Glutamic Acid	nutrient stimulation
PM05	A01	-79	Negative Control	nutrient stimulation
PM05	B08	-79	L-Phenylalanine	nutrient stimulation
PM05	C04	-79	L-Tryptophan	nutrient stimulation
PM05	C02	-78	L-Serine	nutrient stimulation
PM05	D11	-78	Cytidine	nutrient stimulation
PM05	D10	-77	Cytosine	nutrient stimulation
PM05	D12	-77	2`-Deoxy-Cytidine	nutrient stimulation
PM05	E08	-77	D-Pantothenic Acid	nutrient stimulation
PM05	D02	-77	L-Citrulline	nutrient stimulation
PM05	C08	-76	4-Hydroxy L-Proline (trans)	nutrient stimulation
PM05	D09	-76	D,L-Diamino-Pimelic Acid	nutrient stimulation
PM05	A03	-75	L-Alanine	nutrient stimulation
PM05	B02	-75	Glycine	nutrient stimulation
PM05	D07	-75	D-Aspartic Acid	nutrient stimulation
PM05	C01	-75	L-Proline	nutrient stimulation

PHENOTYPE MICROARRAY (PM)- NO	WELL	VALUE	SUBSTRATE	MODE OF ACTION
**Phenotypes Lost(cont'd):**				
PM05	A09	-74	Adenosine-3',5'-Cyclic Monophosphate	nutrient stimulation
PM05	B01	-74	L-Glutamine	nutrient stimulation
PM05	D01	-74	L-Ornithine	nutrient stimulation
PM05	E04	-74	Pyridoxine	nutrient stimulation
PM05	F09	-73	N-Acetyl D-Glucosamine	nutrient stimulation
PM05	E12	-73	2`-Deoxy-Uridine	nutrient stimulation
PM05	F10	-73	Thymine	nutrient stimulation
PM05	F08	-73	D-(+)-Glucose	nutrient stimulation
PM05	E07	-72	b-Alanine	nutrient stimulation
PM05	E09	-72	Orotic Acid	nutrient stimulation
PM05	F06	-72	Hematin	nutrient stimulation
PM05	F04	-72	b-Nicotinamide Adenine Dinucleotide	nutrient stimulation
PM05	F05	-69	d-Amino-Levulinic Acid	nutrient stimulation
PM05	B06	-68	L-Lysine	nutrient stimulation
PM05	G04	-68	p-Amino-Benzoic Acid	nutrient stimulation
PM05	B05	-67	L-Leucine	nutrient stimulation
PM05	A10	-66	Adenine	nutrient stimulation
PM05	G08	-65	Thiamine Pyrophosphate	nutrient stimulation
PM05	F03	-65	Nicotinamide	nutrient stimulation
PM05	G05	-64	Folic Acid	nutrient stimulation
PM05	G01	-64	Oxaloacetic Acid	nutrient stimulation
PM05	F02	-64	Nicotinic Acid	nutrient stimulation
PM05	F11	-64	Glutathione (reduced form)	nutrient stimulation
PM05	E01	-64	Putrescine	nutrient stimulation
PM05	B04	-63	L-Isoleucine	nutrient stimulation
PM05	F07	-63	Deferoxamine Mesylate	nutrient stimulation
PM05	E02	-62	Spermidine	nutrient stimulation
PM05	G03	-61	Cyano-Cobalamine	nutrient stimulation
PM05	G09	-60	Riboflavin	nutrient stimulation
PM05	G02	-60	D-Biotin	nutrient stimulation
PM05	H05	-58	D,L-a-Lipoic Acid (oxidized form)	nutrient stimulation
PM05	H08	-58	Choline	nutrient stimulation
PM05	F01	-58	Quinolinic Acid	nutrient stimulation
PM05	H06	-58	D,L-Mevalonic Acid	nutrient stimulation
PM05	H07	-57	D,L-Carnitine	nutrient stimulation
PM05	H10	-56	Tween 40	nutrient stimulation
PM05	B07	-55	L-Methionine	nutrient stimulation
PM05	G07	-55	Thiamine	nutrient stimulation
PM05	H04	-55	Caprylic Acid	nutrient stimulation
PM05	E05	-50	Pyridoxal	nutrient stimulation
PM12B	B05	-98	Penimepicycline	protein synthesis, tetracycline
PM13B	G03	-116	Cobalt chloride	toxic cation
PM16A	A03,A04	-141	Cefotaxime	wall, cephalosporin

Table 1: Phenotypes gained or lost of RN6390 *hfq* mutant
